# TNRSim — a simulator of truncated oxford nanopore direct RNA long reads for isoform analysis

**DOI:** 10.3389/fbinf.2026.1694068

**Published:** 2026-09-29

**Authors:** Anna Kozlova, Artemiy Sakharov, Svetlana Tarbeeva, Ekaterina Ilgisonis, Elena Ponomarenko

**Affiliations:** Institute of Biomedical Chemistry, Moscow, Russia

**Keywords:** alternative splicing, direct RNA sequencing, isoform analysis, nanopore sequencing, simulation

## Abstract

**Introduction:**

Nanopore sequencing opens up new opportunities in transcriptome research due to the possibility of generating full-length isoform reads. However, currently available simulation software does not fully reproduce read truncation, a key feature of transcriptomic nanopore libraries.

**Methods:**

We developed TNRSim (Truncated Nanopore Reads Simulator), a nanopore transcriptome library simulator designed to reproduce realistic read-length distributions, sequencing errors, and quality scores. We further used simulated datasets with known isoform abundances to benchmark widely used isoform quantification tools.

**Results:**

TNRSim reproduced characteristic read-truncation patterns observed in empirical direct RNA sequencing datasets and provided improvements in read-length distribution, mismatch, and quality-score simulation compared with existing approaches. In a closed-set benchmark of isoform quantification methods, oarfish achieved the highest quantification accuracy among the tested tools.

**Discussion:**

TNRSim provides a framework for realistic simulation of transcriptomic nanopore sequencing data and enables controlled benchmarking of computational methods for isoform-level analysis.

## Introduction

Nanopore sequencing technology provides previously absent opportunities for studying complex transcriptomes: long reads allow to perform isoform level analysis and detection of new isoforms, but there is one nuance. A nanopore sequencer is able to make reads longer than 10 kbp, but in transcriptome libraries, the length of reads is limited by the length of the readable RNA molecules. Fragmentation of mRNA molecules during sample preparation leads to the presence of reads in transcriptome libraries with a missing part at the 5'-end called truncated reads (Sessegolo et al., 2019). The fragmentation degree is the same for all genomic DNA transcripts in the library and it is the characteristic of a library. In libraries with high fragmentation degree truncated reads are predominant; for long isoforms the number of full-length reads is extremely low or may even be zero for low expressed isoforms.

Truncation of reads creates difficulties for isoform level analysis, which should be taken into account when developing programs for transcriptomic analysis. The only currently available simulator of ONT transcriptome libraries, NanoSim (Hafezqorani et al., 2020), generates read length distribution that does not correspond to real libraries (v3.2.3) (Prjibelski et al., 2023). The key idea in the development of TNRSim was to study the read length distribution and to reproduce it correctly.

We use the term *fragmentation* to denote the stochastic per-nucleotide breakage (degradation) of RNA molecules during sample preparation that results in 5′-truncated reads in direct RNA libraries. This differs from intentional enzymatic fragmentation used in some cDNA library protocols or from sequencing artifacts; TNRSim is designed to model the stochastic degradation observed in direct RNA (dRNA) libraries.

## Materials and methods

### Direct RNA nanopore sequencing data

Publicly available direct RNA nanopore sequencing data for Huh7 (PRJNA893571), HepaRG (PRJNA1366635), and HepG2 (PRJNA765908) cell lines were obtained from the NCBI SRA repository. These datasets were originally generated and described in (4, 5, 14). Briefly, the original Huh7 and HepG2 libraries were prepared using the Direct RNA Sequencing Kit (SQK-RNA002, ONT) and sequenced on MinION platform with FLO-MIN106 flow cells. The HepaRG library was generated using the Direct RNA Sequencing Kit (SQK-RNA004, ONT) on a PromethION platform. For the current study, pre-processed “fastq” files were initially downloaded from SRA. Raw “fast5″ files for Huh7 and HepG2 cell lines were retrieved and re-basecalled using the Dorado basecaller (ONT) with standard parameters. The HepaRG data, which were originally basecalled with Dorado, were utilized as available.

### Characterization and simulation of the read length distribution

Often in complex transcriptomes such as the human transcriptome, the shape of the read length distribution of truncated reads is distorted by peaks corresponding to full-length reads of short isoforms, as well as by the presence of multiple isoforms contributing to the same genomic alignment. As a result, direct estimation of fragmentation parameters from the global shape of the truncated read length distribution is challenging.

To rigorously evaluate the suitability of the geometric assumption under these conditions, we implemented a formal model comparison framework that fits candidate distributions: geometric, exponential, log-normal, and discrete Weibull, to per-transcript read length data using maximum likelihood estimation. Model selection is performed using the Akaike Information Criterion (AIC) and Kolmogorov–Smirnov goodness-of-fit tests, with numerical safeguards to ensure stability at large sample sizes. The geometric distribution is retained as the default parameterisation due to its direct biological interpretability (per-nucleotide break probability), computational efficiency, and statistical equivalence to the exponential model for integer-valued read lengths upon rounding. All comparisons are conducted on transcripts with ≥300 aligned reads to ensure reliable parameter estimation, and the resulting fit statistics are reported in Supplementary Table S1.

We estimate the read length distribution parameters using the relationship between the number of full-length reads and the number of truncated reads observed at a selected length. Let.

-
$N_{m}$ denote the total number of RNA molecules for a selected isoform,
-
$N_{f}$ denote the number of full-length reads, and
-
$N_{t}$ denote the total number of truncated reads for that isoform.

By definition, the relationship between these quantities is given by Equation 1:

$$N_{m}=N_{t}+N_{f}$$ (1)

$$N_{f}=N_{m}-N_{t}$$ (2)

Let $n_{l}$ denote the number of truncated reads of length *l*. We select $n_{l}$ at a length immediately preceding the full-length peak, where the distribution is least affected by distortions.

To model fragmentation, we assume that each nucleotide position breaks independently with the same probability *p* (per-nucleotide break probability). Under this assumption, the length of a truncated read follows a geometric distribution: the probability that the first break occurs after exactly *l* nucleotides is *p*(*1−p*)^*l*-1^. Therefore, the expected number of truncated reads of length *l* is

$$n_{l}=p*N_{m}*{\left(1-p\right)}^{l}$$ (3)

The total number of truncated reads for an isoform of length *L* is obtained by summing over all possible truncated read lengths:

$$N_{t}=\sum_{l=1}^{L}p*N_{m}*{\left(1-p\right)}^{l}$$ (4)

A full-length read is generated when no break occurs in the first *L* nucleotides, which happens with probability (*1−p*)^*l*^. Therefore, the expected number of full-length reads is

$$N_{f}=N_{m}*\left(1-p*\sum_{l=1}^{L}{\left(1-p\right)}^{l}\right)$$ (5)

Therefore, Formula 3 relates the number of reads at a selected length to the number of RNA molecules for the selected isoform and the fragmentation degree. Formula 4 expresses the number of truncated reads in terms of the total number of RNA molecules for selected isoform, length of the isoform and the fragmentation degree. Formula 5, derived from Formulas 2 and 4, relates the number of full-length reads to the total number of RNA molecules for selected isoform, the length of the isoform, and the fragmentation degree.

From Formulas 3 and 5, a system of equations with two unknown quantities (*N*_*m*_ and *p*) can be obtained. The number of reads at a selected length and the number of full-length reads are known quantities (determined from the read length distribution). Solving this system of equations allows us to obtain the values of unknown quantities. The length distribution of truncated reads follows the geometric distribution.

To quantify RNA fragmentation across datasets, we analyzed aligned reads from BAM files generated for each downloaded library. Read lengths were extracted using samtools view, and empirical length distributions were constructed for isoforms with >300 aligned reads. Under the geometric model, RNA fragmentation is characterised by a single parameter *p*, representing the per-nucleotide break probability (see Formula 3). This parameter serves directly as the quantitative measure of fragmentation degree: higher values of *p* indicate greater susceptibility to fragmentation, resulting in shorter read length distributions. Fragmentation degree (f) was estimated by fitting an exponential decay model to the observed read length histograms for each transcript in a given library using non-linear least squares optimization. Libraries were then ranked by their estimated f parameter: those with f values above the cohort median were classified as “high-fragmentation” (f = 0.001), while those below were classified as “low-fragmentation” (f = 0.0004).

### Characterization and simulation of errors

A nanopore sequencer makes three types of errors: mismatches, insertions and deletions. The length of errors is sampled from a mixed distribution: Poisson-Geometric for mismatches and Weibull-Geometric for insertions and deletions (Yang et al., 2017). The length of match regions are selected using weighted random choice. The parameters of mixed distributions and weights for matching regions are determined at the characterization stage.

To correctly reproduce the nucleotide fixity the mismatch position shift algorithm is used. It shifts position of nucleotide replacement in accordance with the probability of mismatch on the current nucleotide type: the higher the fixity of the nucleotide, the higher the probability that the mismatch will be shifted to the next or previous nucleotide. In addition, the replaced nucleotide is changed by another that is selected in accordance with the transition probability of the replaced nucleotide to another three nucleotides. These transition probabilities are determined at the characterization stage. To quantify the agreement between simulated and empirical mismatch patterns, we computed the Spearman rank correlation coefficient between the 12 off-diagonal elements of the TNRSim substitution matrix and the corresponding values derived from aligned real reads.

The model of homopolymer simulation is similar to that of NanoSim: the length of the homopolymer in the read is sampled from a normal distribution; the parameters of this distribution depend on the length of the homopolymer in the reference. Description of handling with unaligned regions on the ends of reads and Q-score characterization and simulation is in Supplementary Note S1.

### Data simulation for isoform analysis

To benchmark isoform quantification tools under controlled conditions, we designed a simulation framework with known ground truth expression levels. Three independent expression profiles were generated, each comprising approximately 1,700 isoforms with assigned read counts drawn uniformly from the range 1–1,100. Using TNRSim, we simulated six FASTQ datasets from these profiles: three replicates with high fragmentation degree (f = 0.001) and three with low fragmentation degree (f = 0.0004), preserving the underlying isoform abundances in each case.

Simulated reads were aligned using minimap2 (v2.28) in two modes (Sessegolo et al., 2019): genome alignment to GRCh38. p14 with splice-aware parameters (-ax splice--junc-bed -uf -k14), and (Hafezqorani et al., 2020) transcriptome alignment to GENCODE v46 with the -ax map-ont preset. Isoform abundances were then estimated using six quantification tools: Salmon quant (v1.10.0) (Hu et al., 2021), oarfish (v0.7.0) (Tang et al., 2020), LIQA (v1.2.0) (Soneson et al., 2019), IsoQuant (v3.6.3) (Prjibelski et al., 2023), FLAIR (2.0) (Mikheenko et al., 2022) and Isosceles (v0.2.1) (Kabza et al., 2024) programs. Basic commands for isoform quantification are given in Supplementary Note S2.

To evaluate accuracy, we computed the Spearman rank correlation between the predicted isoform expression values and the known ground truth abundances for each tool and fragmentation condition. Statistical significance of correlation coefficients was assessed using exact permutation testing with 1000 permutations.

### Statistical analysis of the dependence of fragmentation on transcript length

To analyze the factors affecting the acquisition of full-length reads, only transcripts with a total number of mapped reads (read count) of at least 5 were used. A read was considered full-length if it covered ≥95% of the annotated transcript’s length. For each transcript, a binary variable indicating the presence of at least one such read was defined.

To test the statistical significance of the influence of length and read count, binomial logistic regression (Bernoulli logistic regression) was applied. The model predicts the probability *p* of having at least one full-length read as a function of the predictors (Formula 6):

$$logit\left(p\right)=\beta_{0}+\beta_{1}*\log_{10}\left(length\right)+\beta_{2}*\log_{10}\left(read\;count+1\right)$$ (6)

The model was implemented using the Logit function from the statsmodels library (v.0.14.6) for Python 3. The significance of the coefficients was assessed using the Wald test. This model was chosen due to the binary nature of the target variable and the need to evaluate the influence of continuous predictors on the log-odds of the event.

### Performance

TNRSim could use multiple threads and its performance is suitable for use on laptops. For example, a library containing 500,000 reads (580M bases) was simulated on an ultrabook with an AMD Ryzen 7 4700U processor using 6 cores in 27 min (OS Ubuntu 20.04.06 LTS).

## Results

### TNRSim produces reads with different fragmentation degree

To demonstrate the capabilities of the new simulator, we compare several publicly available direct RNA sequencing datasets and data generated by TNRSim and NanoSim. By comparing real and simulated libraries, we show that TNRSim is similar to NanoSim in error model and has higher fidelity in some other respects such as read length distribution and Q-score generation.

For transcriptomic nanopore libraries, the length distribution plays an important role in terms of data suitability for isoform analysis. The correct reproduction of this parameter is a key aspect in the process of transcriptome libraries simulation. Prior work has noted that transcriptome read-length profiles are complex (Hafezqorani et al., 2020). NanoSim approaches this by reproducing the global read-length distribution observed in a library. Indeed, the general read length distribution is complex, as it is formed by thousands of isoforms with different lengths and expression levels.

Analysis of read-length distributions for individual genes revealed a characteristic pattern: a prominent peak of short, 5′-truncated reads followed by an exponential decay toward longer lengths (Figure 1). This pattern is consistent with a simple mathematical model in which each nucleotide position has an equal probability of breakage during library preparation, resulting in a geometric distribution of truncated read lengths for individual isoforms.

**FIGURE 1:**
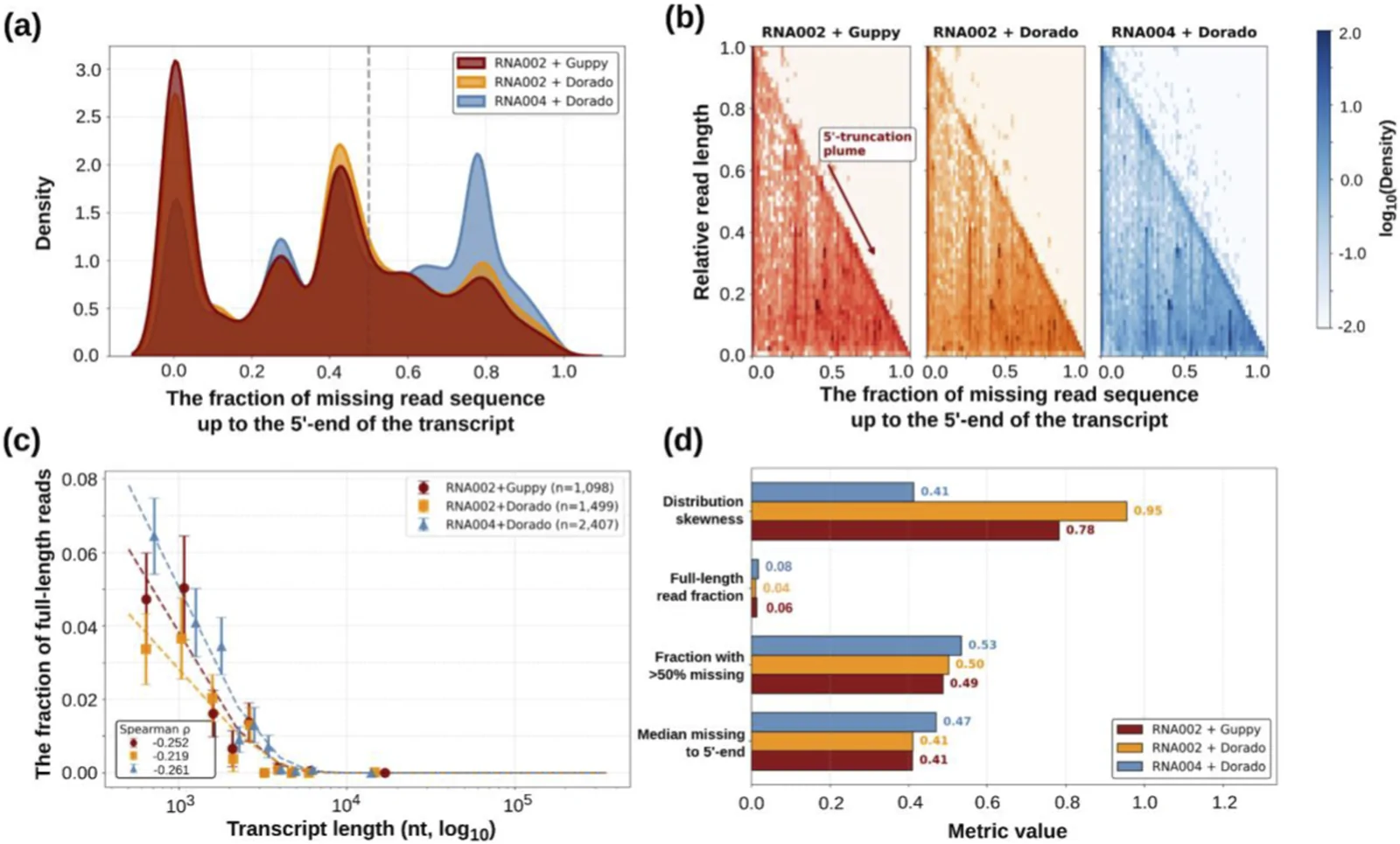
Library characteristics across direct RNA sequencing datasets. **(a)** Density distributions of the fraction of missing read sequence up to the 5′-end of the transcript for three datasets: RNA002+Guppy (HepG2, PRJNA765908), RNA002+Dorado (HepG2), and RNA004+Dorado (HepaRG, PRJNA1366635). Vertical dashed line indicates 50% missing sequence threshold. **(b)** Two-dimensional density heatmaps showing the relationship between relative read length and fraction of missing 5′sequence. The diagonal “5′-truncation plume” (indicated by arrow in left panel) represents reads truncated at various positions along the transcript. Color scale shows log_10_(density). **(c)** Fraction of full-length reads as a function of transcript length (log scale) for the three datasets. Points show empirical values with binomial confidence intervals; dashed lines show exponential decay fits. Spearman correlation coefficients (ρ) are shown in the inset. **(d)** Summary metrics comparing fragmentation characteristics across datasets: distribution skewness, full-length read fraction, fraction of reads with >50% missing sequence, and median fraction missing to 5′-end. Values are shown for RNA002+Guppy (red, n = 1,098 transcripts), RNA002+Dorado (orange, n = 1,499), and RNA004+Dorado (blue, n = 2,407).

We conducted a formal comparison of the geometric distribution against exponential, log-normal, and Weibull alternatives using Akaike Information Criterion on the complete read length datasets from two independent libraries (HepG2_4_s: n = 1,499,762; Huh7_3_s: n = 842,525, see Supplementary Table S1). The exponential distribution yielded the lowest AIC values (ΔAIC = 1600 relative to the geometric model); however, we note that geometric and exponential distributions belong to the same family of memoryless processes, differing solely in their domain of definition (discrete versus continuous). For the purpose of simulating integer-valued read lengths, these two parameterisations are statistically equivalent: sampling from an exponential distribution followed by rounding produces outputs indistinguishable from direct geometric sampling, as confirmed by two-sample Kolmogorov–Smirnov tests (p > 0.05). More flexible models (log-normal, Weibull) could not be reliably fitted due to numerical instability in log-likelihood computation at sample sizes exceeding 10^6^ observations, resulting in infinite AIC values and indicating overparameterisation for the fragmentation process under study. The geometric parameter *p* retains direct biological interpretability as the per-nucleotide break probability and ensures computational efficiency of the simulator without compromising the fidelity of aggregate error statistics.

Figure 1a shows the distribution of missing sequence fraction across three datasets sequenced with different chemistries (RNA002 and RNA004) and basecallers (Guppy and Dorado). All datasets exhibit a bimodal pattern with peaks near 0% (full-length reads) and 40%–50% (truncated reads), though the relative proportions differ. The RNA004+Dorado dataset shows a higher proportion of reads missing >50% of the 5′end (0.53) compared to RNA002 datasets (0.49–0.50), suggesting that the 5′-truncation phenomenon persists in newer sequencing chemistries.

The 2D density plots in Figure 1b reveal the characteristic “5′-truncation plume” — a diagonal band extending from full-length transcripts (top right) to short fragments (bottom left). This pattern is visible across all three protocol variants, confirming that truncation occurs throughout the transcript body rather than at specific positions. The plume is most pronounced in RNA002+Guppy data and appears slightly attenuated in RNA004+Dorado, consistent with improvements in library preparation and basecalling accuracy.

To validate the exponential decay assumption, we examined the relationship between transcript length and the fraction of full-length reads (Figure 1c). As predicted by the model, longer transcripts show progressively lower probabilities of being sequenced in full. Spearman correlation analysis revealed significant negative correlations across all datasets (ρ = −0.219 to −0.261, p < 0.001). Binomial logistic regression confirmed that transcript length is a highly significant predictor of full-length read detection (β = −5.58, p < 0.001), while expression level showed no significant effect (p = 0.583) in our dataset.

Figure 1d summarizes key fragmentation metrics across the three protocols of sample preparation and data pre-processing. The distribution skewness is highest for RNA002+Dorado (0.95), intermediate for RNA002+Guppy (0.78), and lowest for RNA004+Dorado (0.41), indicating progressively more symmetric length distributions with newer protocols. However, all three protocols show substantial fractions of truncated reads (median missing 41%–47% of the 5′end), demonstrating that fragmentation remains a dominant feature requiring accurate modeling.

Despite advances in nanopore simulation, neither TNRSim nor NanoSim fully recapitulates the characteristic 3′-end coverage bias observed in real direct RNA sequencing data (Supplementary Figure S1). While TNRSim reproduces the general trend of increasing coverage toward the 3′end through its exponential length distribution model, both simulators fail to capture the sharp coverage increase in the terminal 10%–20% of transcripts that is consistently observed in empirical data, suggesting that additional biological or technical factors beyond simple fragmentation contribute to the 3′-end enrichment pattern.

We compared read-length distributions from real direct RNA sequencing data with simulations from TNRSim and NanoSim of APOB transcript (APOB-201) in 3 cell lines (Figure 2). TNRSim successfully reproduces the characteristic features of empirical distributions—the asymmetric plume of 5′-truncated reads and, where present, peaks corresponding to full-length transcripts. This pattern extends transcriptome-wide. As shown on Supplementary Figure S2 read length distributions for representative isoforms of five randomly selected genes (APOB, AHSG, MT-ND4, MT-RNR2, AFP), TNRSim consistently captures the exponential decay and long tail of truncated reads. NanoSim produces more symmetric distributions with fewer extreme outliers.

**FIGURE 2:**
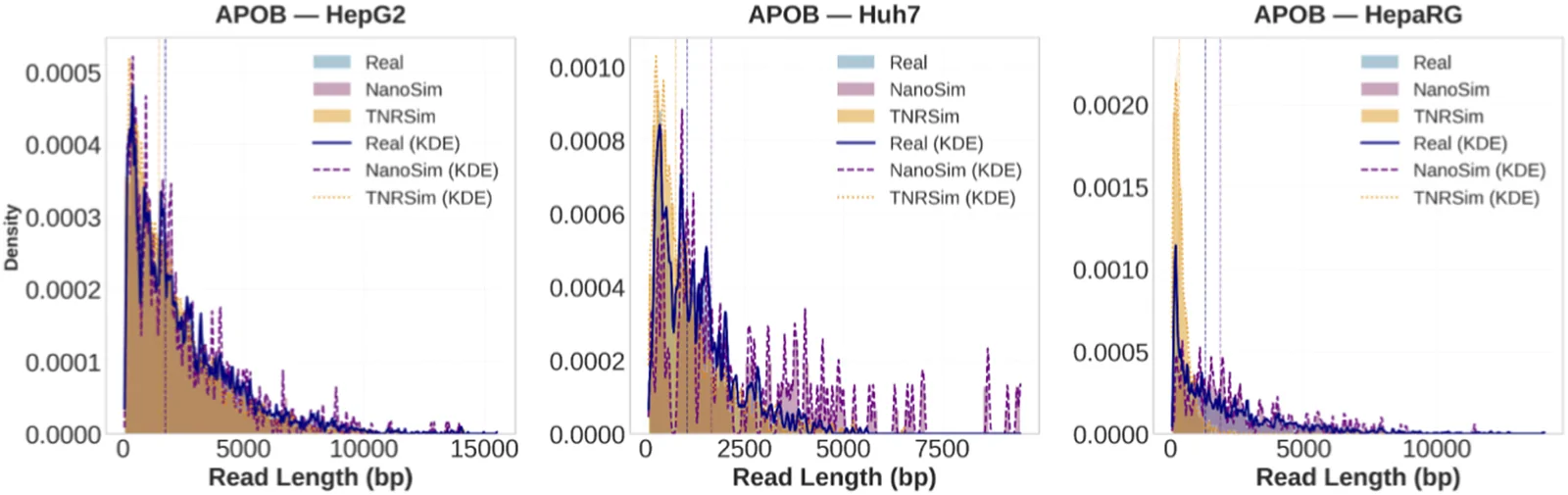
Read-length distributions: comparison of real data and simulations. Density plots of read lengths for the APOB-201 isoform in 3 cell lines: HepG2, Huh7, and HepaRG. Each panel compares real sequencing data (blue) with simulations from NanoSim (purple) and TNRSim (orange). Solid lines show kernel density estimates (KDE); dashed lines indicate median read lengths. Vertical dotted lines mark the annotated transcript length. Kolmogorov-Smirnov test statistics comparing real vs. simulated distributions: HepG2 (TNRSim: D = 0.12, NanoSim: D = 0.18), Huh7 (TNRSim: D = 0.09, NanoSim: D = 0.15), HepaRG (TNRSim: D = 0.11, NanoSim: D = 0.14); all p < 0.001.

For APOB in HepG2 cells, both real data and TNRSim simulations show a sharp peak at short lengths (∼500 bp) followed by exponential decay, with no distinct full-length peak (the APOB transcript is ∼14 kb, making full-length reads rare at the observed fragmentation level). NanoSim produces a similar overall shape but with less pronounced truncation. In Huh7 cells, where fragmentation is higher, both real and TNRSim data show broader distributions with more reads in the 2–5 kb range. For HepaRG, sequenced with RNA004 chemistry, the distribution is more compact with a sharper peak near 1 kb, a pattern captured by TNRSim.

Kolmogorov-Smirnov tests reveal that all simulators produce distributions statistically different from real data (p < 0.001 for all comparisons), reflecting the inherent complexity of biological variation. However, TNRSim shows better qualitative agreement with the characteristic truncation patterns, particularly for highly fragmented libraries.

To assess the factors influencing the detection of full-length reads, we analyzed 35,716 transcripts with an expression level of at least 5 reads. For statistical validation, a binomial logistic regression was employed, where the binary target variable was the presence of at least one full-length read, and the predictors were the logarithms of transcript length and its expression level (Supplementary Note S3 and Supplementary Figure S3). The model revealed a highly significant negative influence of transcript length on the log-odds of detecting a full-length read (coefficient β = −5.58, p < 0.001), while the influence of the expression level was statistically insignificant (p = 0.583). These results indicate that transcript length is the dominant factor associated with the probability of observing full-length reads in our dataset, whereas transcript expression level does not show a detectable effect under this model.

The fragmentation degree is a numerical parameter that is set at the simulation stage in the TNRSim program, which allows you to simulate data from libraries with a high fragmentation degree, as well as with a low one. TNRSim determines the fragmentation degree at the characterization stage. There is also an option to determine only the fragmentation degree without full characterization of the library, which can be used for quality control of nanopore dRNA libraries.

### TNRsim reproduces sequencing errors similar to real data

Nanopore sequencers produce reads with a high error rate (Laver et al., 2015). For correct testing of data processing algorithms, it is necessary to correctly reproduce the errors of the sequencing platform. The TNRSim error model, like NanoSim, is based on a statistical characterization of real reads. The result obtained is also similar to NanoSim and TNRSim and corresponds to real data in terms of base calling events length distribution (Supplementary Figure S4). Supplementary Figure S5 summarizes indel-rate distributions across transcripts, while mismatch substitutions are evaluated separately through the substitution-matrix analysis. For indel rates, TNRSim produces distributions with medians comparable to real data across transcripts, though both simulators show substantial variability that only partially captures the empirical error patterns.

We found that the type of the next error is independent of the current error, so the process of choosing the next error is not a Markov process, the type of error is chosen randomly according to the frequency of occurrence of errors of different types. It was also noted that after insertion and deletion, a single nucleotide mismatch follows with a probability of 0.08 and 0.07 respectively (Supplementary Figure S6). This feature was taken into account in the TNRSim error model.

TNRSim brings improvements to mismatch simulation in terms of nucleotide fixity. Mismatches occur with different probabilities on different nucleotides, for example, the most common mismatch error is replacing A with G, and the least common is changing G for C. The TNRSim error model takes into account the different probability of transitions from one base to another, thereby achieving a higher simulation fidelity. The Spearman correlation coefficient between the TNRSim substitution matrix and the real data is 0.95 while that of NanoSim is 0.70, since mismatches occur equiprobable in all four nucleotides and replacing nucleotides are selected randomly with equal probability.

The lag-1 autocorrelation of the Q-score in dRNA reads Oxford Nanopore was 0.58. This value indicates a positive spatial correlation where low quality or an error at one position influences the quality assessment of nearby nucleotides. This feature was taken into account when implementing the data simulation model in the TNRSim program. To model this dependency explicitly, TNRSim employs a first-order autoregressive (AR(1)) process for Q-score generation (Supplementary Note S1, formula 7). Figure 3b shows the distribution of the Q-score in 16 305 match-sections of length 20 b. p. Observed in real HepG2 cell line library. The distribution on the heatmap has a flipped U letter shape (marked by yellow) that means that near errors, the quality is lower than in the middle of a match region. This “inverted U″ pattern emerges because the simulated Q-score decreases locally near a mismatch boundary and then autocorrelatively recovers to a higher baseline towards the center of a matching segment. Figure 3a shows that, in our implementation, NanoSim samples per-base Q-scores from a truncated log-normal distribution and therefore does not reproduce the empirically observed lag-1 autocorrelation; by contrast, TNRSim implements an autocorrelation model that captures this feature. The resulting U-shape is therefore not a periodic artifact but a direct visualization of local quality decay and recovery, accurately captured by the autoregressive process. Thus, the U-shaped distribution is not an artifact but a key validation that TNRSim captures the autocorrelated nature of sequencing errors, which is missed by other simulators. Q-score model in TNRSim simultaneously reproduces the autocorrelation feature and distribution shown in Figures 3a,b.

**FIGURE 3:**
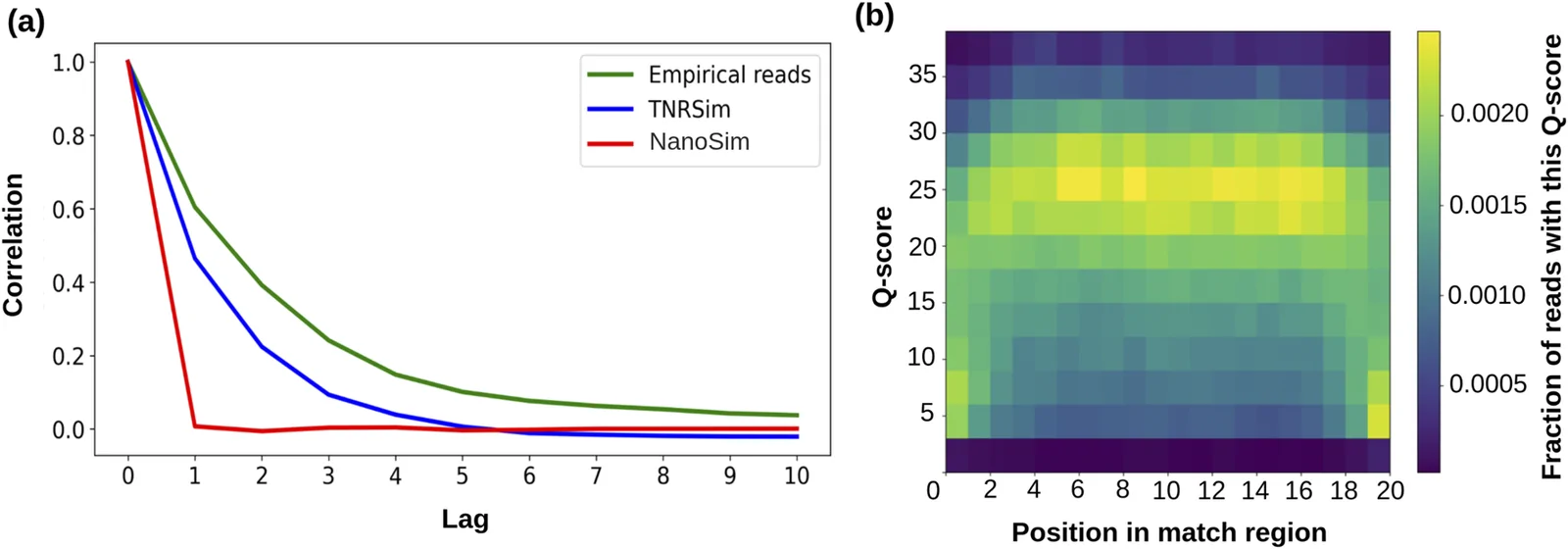
**(a)** Q-score autocorrelation. **(b)** 2D histogram of the distribution of Q-scores observed in match regions in the real HepG2 cell line library (empirical data).

### TNRSim for testing isoform quantification tools

The accurate quantification of isoforms is crucial for understanding gene expression regulation, disease mechanisms, and biomarker discovery. Recent studies demonstrated the key role of changes in the expression profile of isoforms in cancer cells. This phenomenon has come to be called “isoform switching” (Kahraman et al., 2020). With the increasing volume of RNA-seq data, computational tools for isoform analysis play a key role in extracting meaningful biological insights. However, differences in algorithmic approaches can lead to variability in results across different software solutions. Thus, rigorous testing and benchmarking of these tools are essential to ensure accuracy, reproducibility, and reliability in isoform quantification.

To test the effect of the fragmentation degree on the accuracy of isoform analysis, six simulated libraries containing approximately 1700 isoforms (three with low fragmentation degree and three with high fragmentation degree) were used. The most popular programs for isoform quantification in direct RNA nanopore sequencing data such as Salmon quant, oarfish, LIQA, IsoQuant and FLAIR were tested. These tools were selected to represent distinct and widely used methodological approaches to long-read isoform quantification, including probabilistic read assignment, splice graph-based reconstruction, and error-correction–driven transcript collapsing. The selection was limited to methods that explicitly support nanopore data, are actively maintained, and are compatible with our simulation and analysis pipeline; other long-read quantification tools were therefore not included in this benchmark.

While LIQA, IsoQuant and FLAIR using genome alignments, Salmon quant and oarfish dealt with transcriptome alignments. Each of these tools employs distinct algorithmic strategies. For isoform abundance estimation, Salmon quant, LIQA and oarfish apply Expectation-Maximization (EM) algorithm ensuring a probabilistic distribution of reads across isoforms while accounting for sequencing biases (Patro et al., 2017; Jousheghani and Patro, 2024; Hu et al., 2021). Initially, Salmon quant was developed to work with short reads, but starting with version 1.5.0, an option that supports working with nanopore sequencing data was added. IsoQuant utilizes a splice graph-based approach for reconstructing full-length isoforms from long-read sequencing data (Prjibelski et al., 2023). The algorithm constructs a probabilistic model that estimates isoform structures and their relative expression levels. FLAIR algorithm applies error correction and junction filtering to refine transcript models and remove low-confidence splicing events (Tang et al., 2020). It further employs a transcript collapsing strategy, reducing redundancy in isoform annotations and improving quantification accuracy.

In addition, we included Isosceles, which implements a distinct splice graph–based framework combined with Transcript Compatibility Counts (TCC) for isoform quantification. By modeling read compatibility with transcript structures rather than performing direct read-to-isoform assignment, Isosceles represents a state-of-the-art approach that has demonstrated high quantification accuracy in recent long-read benchmarking studies. The inclusion of Isosceles extends the methodological diversity of our benchmark and enables a more comprehensive comparison of current long-read isoform quantification strategies. The resulting precision and recall values are presented in Table 1.

**TABLE 1:** Precision and recall values of isoform identifications by quantification programs on simulated data.

Program	Metric	Fragmentation degree					
		*Low*			*High*		
		*1*	*2*	*3*	*1*	*2*	*3*
Salmon quant	Precision	0.81	0.81	0.79	0.72	0.72	0.70
	Recall	0.74	0.72	0.71	0.75	0.74	0.73
Oarfish	Precision	0.91	0.91	0.91	0.78	0.78	0.76
	Recall	0.99	0.99	0.99	0.95	0.95	0.95
LIQA	Precision	0.50	0.46	0.47	0.43	0.39	0.39
	Recall	0.27	0.24	0.24	0.23	0.21	0.21
IsoQuant	Precision	0.86	0.87	0.84	0.80	0.79	0.79
	Recall	0.35	0.33	0.34	0.27	0.26	0.27
FLAIR	Precision	0.46	0.49	0.49	0.69	0.72	0.70
	Recall	0.19	0.21	0.20	0.26	0.29	0.28
Isosceles	Precision	0.49	0.49	0.49	0.48	0.49	0.49
	Recall	0.25	0.24	0.24	0.21	0.21	0.20

Because the simulated libraries contain only annotated transcripts, these metrics assess isoform quantification and the detection of known isoforms under controlled conditions, rather than *de novo* alternative splicing discovery. Crucially, this analysis was explicitly designed to evaluate the qualitative accuracy of splice isoform identification, that is, the ability of each method to correctly detect the presence or absence of isoforms, rather than to assess the accuracy of transcript abundance estimates. This analysis was designed to assess the qualitative accuracy of splice isoform identification, formulated as a classification task (isoform detected vs. not detected). Accordingly, precision and recall were used as metrics of isoform detection performance under varying degrees of read fragmentation.

Among the tested methods, oarfish demonstrates superior performance, achieving consistently high precision (0.79–0.93) and recall (0.87–0.97) across both low and high fragmentation levels, indicating robust and sensitive isoform identification. Salmon quant and IsoQuant exhibit comparatively high precision (0.70–0.87) but substantially lower recall, reflecting a conservative identification strategy that prioritizes specificity over completeness of isoform recovery. FLAIR shows moderate precision (0.46–0.72), with a noticeable improvement under conditions of high fragmentation, suggesting robustness to read degradation but limited sensitivity in recovering the full set of expressed isoforms.

LIQA and Isosceles display consistently lower recall values across all conditions. LIQA exhibits both low precision (0.43–0.52) and low recall (0.22–0.27), indicating limited sensitivity and specificity for splice isoform identification in fragmented direct RNA nanopore data. Isosceles shows stable but moderate precision (∼0.48–0.49) across fragmentation levels, accompanied by low recall (0.20–0.25). This pattern suggests that the splice graph and Transcript Compatibility Count–based strategy implemented in Isosceles provides robustness to read fragmentation but remains conservative in isoform detection, resulting in reduced sensitivity compared with transcriptome-based EM approaches.

The heatmaps shown on Figure 4 depicting the Spearman correlation coefficients between estimated and true isoform abundances for six isoform quantification tools (Salmon quant, FLAIR, LIQA, Oarfish, IsoQuant and Isosceles) across six independent simulations of reads libraries. In contrast to the precision–recall analysis, which was aimed at assessing the qualitative accuracy of splice isoform identification, this analysis focuses on the quantitative aspect of isoform profiling, namely, the ability of each method to preserve the correct ranking of isoform abundances.

**FIGURE 4:**
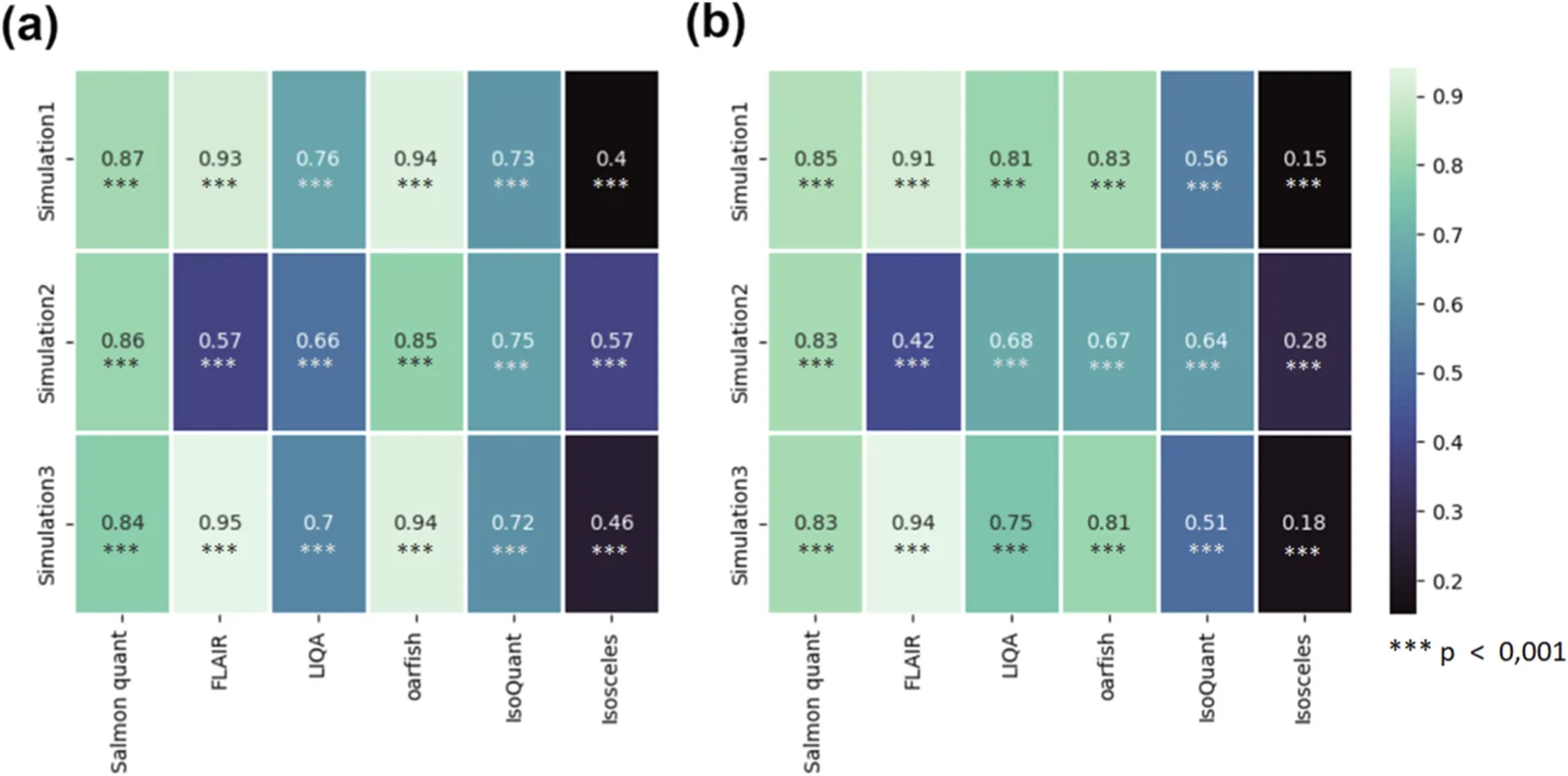
Correlation coefficients between simulated and quantified isoform abundance values across six simulations of libraries with **(a)** low and **(b)** high fragmentation degree.

For libraries with a low fragmentation degree (Figure 4a), oarfish consistently achieves the highest correlation values (0.85–0.94) across all simulations, indicating strong agreement between estimated and true isoform abundance rankings. Salmon quant and IsoQuant also show relatively high correlations (0.70–0.75), suggesting reliable quantitative performance under favorable read integrity conditions. FLAIR demonstrates moderate correlation values (0.57–0.95), with increased variability between simulations, while LIQA exhibits consistently lower correlations (0.66–0.76), indicating reduced accuracy in capturing true abundance relationships. Isosceles shows the lowest correlation values (0.40–0.57), reflecting limited quantitative concordance despite its stable qualitative behavior observed in isoform identification.

A similar trend is observed for libraries with a high fragmentation degree (Figure 4b). Oarfish maintains the highest correlation values (0.81–0.94), confirming its robustness to read degradation. Salmon quant and FLAIR display moderate correlations (0.68–0.91), while LIQA continues to show comparatively weak performance (0.68–0.81). IsoQuant exhibits greater variability under high fragmentation conditions, with correlation coefficients ranging from 0.51 to 0.64 across simulations. Isosceles again yields the lowest correlations (0.15–0.28), suggesting that its splice graph and Transcript Compatibility Count–based framework, while robust for conservative isoform detection, is less effective for accurate quantitative ranking of isoform abundances.

To evaluate whether n = 3 independent simulation replicates provide sufficient statistical resolution, we computed within-tool variability and conducted a post-hoc power analysis using the observed variance structure. The pooled standard deviation across replicates was σ = 0.12, corresponding to a minimum detectable effect of Δr ≥ 0.36 at 80% power (α = 0.05, two-sided t-test). Observed differences between best- and worst-performing tools (Δr = 0.43–0.63) exceeded this threshold, confirming adequate power for discriminating broad performance tiers.

Based on the simulation benchmarks and post-hoc power analysis, oarfish and Salmon quant emerge as the top-performing tools for isoform quantification, consistently achieving the highest correlations with ground-truth abundances across fragmentation conditions. The performance gap between these leading methods and lower-tier tools (Δr = 0.43–0.63) substantially exceeds the minimum detectable effect threshold (Δr ≥ 0.36 at 80% power, n = 3), confirming that broad performance tiers are statistically robust despite the limited number of replicates. While oarfish attains the highest mean correlations, particularly under high fragmentation, Salmon demonstrates superior reproducibility with minimal inter-replicate variability (SD ≤ 0.02), indicating greater stability across stochastic simulation runs. Conversely, fine-grained differences among top-tier tools (Δr < 0.15) fall below the statistical detection threshold, suggesting that precise ordinal rankings should be interpreted cautiously. Collectively, these findings support a tier-based assessment of quantitative accuracy: oarfish and Salmon represent the most reliable options for isoform abundance estimation, with the optimal choice depending on whether the analytical priority is maximal sensitivity to true abundance ranks (oarfish) or consistent reproducibility across technical replicates (Salmon).

The lower quantitative performance of Isosceles under fragmentation likely reflects inherent trade-offs of its TCC-based framework: truncated reads expand equivalence classes, causing the EM algorithm to smooth abundance estimates across many compatible isoforms and thereby reduce rank correlation with ground-truth values (Kabza et al., 2024). This behaviour aligns with the method’s design priority of conservative isoform identification over precise abundance estimation, particularly when fragmentary data provide limited discriminative information.

It is important to note that TNRSim operates as a “closed-set” simulator, generating reads exclusively from known annotated transcripts; consequently, it is well-suited for benchmarking quantification accuracy of reference-based tools but is not designed for evaluating novel isoform discovery methods. Users seeking to assess *de novo* isoform detection capabilities should complement TNRSim benchmarks with orthogonal simulation frameworks or experimental spike-in controls that introduce unannotated transcript variants.

## Discussion

The fragmentation patterns observed in transcriptomic nanopore sequencing reads remain insufficiently characterized, although their presence has been noted in several previous studies. Read fragmentation appears to be a general feature of direct RNA nanopore data and may vary substantially across datasets and experimental protocols. It has been suggested that fragmentation largely arises during sample preparation rather than during sequencing itself, as nuclear and mitochondrial transcripts are sequenced under identical conditions but may experience different biochemical environments prior to library construction. For example, mitochondrial transcripts have been reported to exhibit a particularly high fragmentation degree (Soneson et al., 2019), highlighting the potential influence of RNA localization and handling on read integrity.

Accurate reproduction of read length distributions therefore represents an important challenge for the simulation of transcriptomic nanopore data. Previous work has shown that earlier NanoSim approaches that do not account for transcriptome-specific asymmetries may fail to reproduce empirical read-length profiles (for example, symmetric 5′/3′truncation assumptions are inconsistent with many direct-RNA datasets). Improvements to simulation procedures that better reflect asymmetric truncation and empirical length distributions have been proposed (Prjibelski et al., 2023; Mikheenko et al., 2022). In real transcriptomic nanopore data, read length distributions are typically characterized by the obligatory presence of a broad population of truncated reads and, in some cases, an additional peak corresponding to full-length transcripts, whereas NanoSim-generated data tend to exhibit only discrete peaks. In this context, the present study contributes to a more systematic characterization of transcriptomic nanopore read fragmentation and provides a step toward improving the realism of simulated datasets.

Although data-driven approaches are attractive, we chose a parametric framework because the main objective here is to estimate a library-level fragmentation process from a small number of direct-RNA datasets, with parameters that remain interpretable and transferable across protocols. A machine-learning model would require a much larger, protocol-matched training set and would be harder to validate across different chemistries and library-preparation workflows. This choice is consistent with the long-read simulation literature, where NanoSim is built on statistical characterization of empirical reads, whereas deep-learning simulators such as DeepSimulator were developed for signal-level simulation rather than transcriptome-specific fragmentation (Li et al., 2018). We therefore treat data-driven simulation as an important direction for future work.

This work deepens the understanding of the specifics of nanopore transcriptomic data. It not only improves the testing of isoform analysis algorithms through more accurate simulation, but also expands the theoretical basis for improving existing algorithms. Moreover, the fragmentation degree has been shown to be an important parameter in terms of isoform analysis. TNRSim allows users to determine this value as part of library quality control. It is important to note that the present study and the developed TNRSim tool are focused specifically on the simulation of direct RNA sequencing libraries. Extension of this framework to cDNA protocols (both PCR and PCR-free) would fundamentally require the generation and use of well-characterized cDNA sequencing data from the same biological system as a prerequisite for training accurate bias models, representing a clear direction for future methodological development. Importantly, cDNA and direct RNA (dRNA) nanopore protocols exhibit fundamentally different error and fragmentation characteristics, limiting direct transfer of the present model. In dRNA libraries, sequencing proceeds from the poly(A) tail toward the 5′end, and fragmentation during RNA handling commonly results in a pronounced excess of 5′-truncated reads that can often be approximated by a geometric breakpoint model (Parker et al., 2020). In contrast, cDNA workflows introduce reverse-transcription and frequently PCR-associated biases, including RT misincorporation, template switching, amplification-dependent length bias, and potentially more symmetric or protocol-specific fragmentation patterns Soneson et al., 2019, necessitating dedicated modeling rather than reuse of the dRNA-based framework (Soneson et al., 2019).

It is worth noting that the error model in TNRSim was trained on data obtained using RNA002 chemistry and the Guppy basecaller. Although new protocols (such as RNA004) and algorithms (such as Dorado) demonstrate improved accuracy, fundamental sources of error in nanopore sequencing (such as homopolymer stretches) and the overall noise structure remain relevant. Similarly, certain positional biases prevalent in real data, such as 3′end enrichment, are not yet explicitly captured by the current model. Addressing these nuances in future versions will further enhance the simulator’s fidelity, particularly for applications where coverage uniformity across transcripts is essential. The presented simulation framework is flexible: its core allows for updating error profiles based on new reference data, which opens the way for adapting the tool to future versions of chemistry and software. Thus, TNRSim provides not only a current model but also a basis for validating analysis methods in the context of continuously improving source data quality.

## Data Availability

Publicly available datasets were analyzed in this study. This data can be found here: PRJNA893571, PRJNA1366635, PRJNA765908 (BioProject NCBI).
