# Supplementary material for: TNRSim — a simulator of truncated oxford nanopore direct RNA long reads for isoform analysis
Source: Front Bioinform. 2026 Sep 29;6:1694068. doi: 10.3389/fbinf.2026.1694068 (PMC13658594; doi:10.3389/fbinf.2026.1694068)
Supplement: Supplementary file 1 [file Supplementaryfile1.docx]

**TNRSim – simulator of truncated Oxford Nanopore reads as an instrument for testing algorithms for isoform analysis**

**Supplementary Note S1**

**Unaligned regions**

Both ends of the reads tend to have unaligned sections. Unaligned regions at the 3'-end are divided into 2 groups: short (up to 25 bp in length) and long (75 to 125 bp in length). Short 3’-end unaligned regions are polyA tails. Their length corresponds to the geometric distribution. Long unaligned regions do not have a consensus sequence. We assumed that this sequence was random. Long unaligned regions are simulated as a random sequence with the base abundance determined at the characterization stage. The unaligned regions at the 5'-end of the reads are a random sequence with the nearly equal base abundance and are simulated as a random sequence. The length of the unaligned regions at the 5'-end corresponds to geometric distribution, the parameters of which are determined during the characterization stage.

**Q-score characterization and simulation**

To reproduce the real distribution of quality in match regions TNRSim uses an autoregressive model (Formula 6).

$Q(n)=a*Q(n-1)+c+noise$

(7)

Where *Q(n)* is the quality of the current nucleotide, *Q(n-1)* is the quality of the previous nucleotide, *a* is the autocorrelation coefficient, *c* is a constant value, *noise* is a random value sampled from a bimodal triangular distribution.

The Q-score of the first and the last nucleotides in the match region is sampled from the mismatch quality distribution, since these nucleotides have a quality comparable to the mismatch regions.

**Supplementary Note S2**

**Salmon quant (v1.10.0)**

$../salmon-latest_linux_x86_64/bin/salmon quant -p 10 -t ../path/to/gencode.v46.transcripts.fa -l A --ont -a ../path/to/your.sam -o ../path/to/results/salmon/

**LIQA (v1.2.0)**

$liqa -task quantify -refgene ../path/to/anno.refgene -out ../path/to/results/liqa -max_distance 20 -f_weight 1 -bam ../path/to/your.bam

**oarfish (v0.7.0)**

$oarfish -j 16 -a ../path/to/your.bam -o results_oar --filter-group no-filters --model-coverage

**IsoQuant (v3.6.3)**

$python3 ../IsoQuant-3.6.3/isoquant.py --reference ../path/to/GRCh38.p14.genome.fa --genedb ../path/to/gencode.v46.basic.annotation.gtf --bam ../path/to/your.bam --data_type nanopore -o ../path/to/results

**FLAIR (v2.0)**

$bam2Bed12 -i ../path/to/your.bam > ../path/to/your.bed12

$flair correct -q ../path/to/your.bed12 -f ../path/to/gencode.v46.basic.annotation.gtf -g ../path/to/GRCh38.p14.genome.fa --output ../path/to/results/flair

$flair collapse -g ../path/to/GRCh38.p14.genome.fa --gtf ../path/to/gencode.v46.basic.annotation.gtf -q ../path/to/all_corrected.bed -r ../path/to/your.fastq --stringent --check_splice --generate_map --annotation_reliant generate

$flair quantify -r ../path/to/your_reads_manifest.tsv -i ../path/to/collapse.isoforms.fa --output ../path/to/result

**Supplementary Note S3**

```
Logit Regression Results
==============================================================================
Dep. Variable:        has_full_length   No. Observations:                35716
Model:                          Logit   Df Residuals:                    35713
Method:                           MLE   Df Model:                            2
Date:                Fri, 19 Dec 2025   Pseudo R-squ.:                  0.6410
Time:                        20:04:55   Log-Likelihood:                -1473.9
converged:                       True   LL-Null:                       -4106.0
Covariance Type:            nonrobust   LLR p-value:                     0.000
==================================================================================
                     coef    std err          z      P>|z|      [0.025      0.975]
----------------------------------------------------------------------------------
const             16.0878      0.437     36.796      0.000      15.231      16.945
log_length        -5.5808      0.141    -39.623      0.000      -5.857      -5.305
log_expression     0.0352      0.064      0.549      0.583      -0.090       0.161
==================================================================================
```

**Table S1.** Model selection statistics for candidate fragment length distributions evaluated on HepG2 and Huh7 libraries.

| **Library** | **The Number of Reads** | **Distributions Akaike Information Criterion (AIC)** | | | |
| --- | --- | --- | --- | --- | --- |
|  |  | *Geometric* | *Exponential* | *Log-normal* | *Weibull* |
| HepG2 | 1499762 | 23477879.78 | 23476253.91 | inf | inf |
| Huh7 | 842525 | 12786796.83 | 12785637.02 | inf | inf |

**Figure S1.** Coverage profiles along transcripts: comparison of real data and simulations. Normalized coverage profiles across five genes (APOB, AHSG, MT-ND4, MT-RNR2, AFP) in three datasets: HepG2 (left column, RNA002+Guppy), Huh7 (center column, RNA002+Dorado), and HepaRG (right column, RNA004+Dorado). Each panel shows coverage as a function of transcript position, comparing real sequencing data (solid lines) with simulations from NanoSim (dashed lines) and TNRSim (dotted lines). Coverage is normalized to the maximum value per transcript. The x-axis shows position in base pairs; note the different scales for long (APOB, ~14 kb) and short transcripts (<2 kb). Real data exhibit characteristic coverage patterns including 3′-end enrichment and position-specific variations, while both simulators show varying degrees of success in recapitulating these features.

**Figure S2.** Read length distributions across transcripts: comparison of real data and simulations. **(a)** Box plots showing read length distributions for five genes (APOB, AHSG, MT-ND4, MT-RNR2, AFP) in real sequencing data (pink), TransNanoSim simulations (brown), and TNRSim simulations (green) **(b)** Violin plots displaying the overall read length density distributions aggregated across all transcripts for Real, NanoSim, and TNRSim datasets. The distributions reveal characteristic differences in how each simulator reproduces the empirical read length patterns, with TNRSim capturing the exponential decay and NanoSim producing more uniform distributions.

**Figure S3.** Factors influencing full-length read detection. **(a)** Probability of detecting at least one full-length read as a function of isoform length (log₁₀ scale). Red dots show empirical probabilities with standard deviation bars across 35,716 transcripts; the dashed red line shows the exponential decay model fit (R² = 1.00). The probability decreases sharply with increasing transcript length, confirming that longer transcripts are progressively less likely to be sequenced in full. **(b)** Probability of full-length read detection as a function of expression level (log₁₀ number of reads). The flat relationship indicates that transcript abundance has minimal effect on the probability of observing full-length reads, consistent with the non-significant regression coefficient (p = 0.583). Together, these results validate that transcript length, rather than expression level, is the dominant determinant of full-length read detection in direct RNA sequencing.

**Figure S4.** Benchmarking TNRSim and Trans-NanoSim error model on the direct RNA dataset.

**Figure S5.** Error rate comparisons across transcripts. Box plots showing insertion rate (left), and deletion rate (right) per read for five transcripts (APOB, AHSG, MT-ND4, MT-RNR2, AFP) in real sequencing data (pink), Trans-NanoSim simulations (brown), and TNRSim simulations (green). Both Trans-NanoSim and TNRSim reproduce insertion and deletion errors with varying degrees of fidelity; TNRSim shows median indel rates comparable to real data across most transcripts, while Trans-NanoSim tends to produce slightly higher insertion rates. Boxes indicate interquartile range, horizontal lines show medians, and whiskers represent the full range of observed values.

**Figure S6.** Markov process graphs. Markov-chain model fitted to the full read error walk (states: match, mis, ins, del). Nodes represent error states and edges show transition probabilities inferred by maximum-likelihood fitting; edge color/width visualizes probability magnitude. The model likelihood of observing the example short error subsequence is 0.0012.
